# The parents’ internet use and children’s extracurricular tutoring class participation

**DOI:** 10.1038/s41598-024-62525-x

**Published:** 2024-05-21

**Authors:** Kunpeng Zhang, Tongyang Liu, Dong Xue, Maishou Li

**Affiliations:** School of Economics, Henan University, Kaifeng, 475000 China

**Keywords:** Internet, Extracurricular tutoring class, Social interaction, Educational expectations, CFPS, Environmental sciences, Environmental social sciences

## Abstract

The educational burden from extracurricular tutoring class has become a pressing social issue in China. This study used data from the China family panel studies (CFPS) in 2014, 2016, and 2018 to empirically analyze the impact of Internet usage on children’s participation in extracurricular tutoring class. There are many factors that influence parents’ decisions to enroll their children in extracurricular tutoring class. These factors include family income status, the level of importance parents place on their children’s education, the marginal returns on educational investment, academic pressure, etc. However, in today’s digitalized society, the widespread use of the internet will also become an important influencing factor in parents’ decisions regarding educational investment. The study finds that, parents by using the Internet significantly increase the probability of enrolling their children in extracurricular tutoring class. Through mechanism regression analysis, it is concluded that internet usage has a positive influence on parents enrolling their children in extracurricular tutoring class by increasing the frequency of social interaction and raising parents’ educational expectations for their children. Based on the empirical results, the following policy suggestions were proposed: 1. Schools should establish a more comprehensive after-school education service system to improve the engagement of students in compulsory education; 2. The government can enhance the accessibility and optimization of educational resources by increasing investment in education, improving the quality of in-school education, and optimizing the management and supervision of extracurricular tutoring class. This ensures that students can access high-quality educational services.

## Introduction

Education, as a core element of human capital, plays a critical role in increasing individual well-being, promoting social mobility, and interrupting the intergenerational transmission of poverty. Investment in education is pivotal in stymieing the intergenerational transmission of poverty^1–4^). However, the escalating trend of commercialization in China’s educational landscape in recent years has insidiously transmuted what was once a benign investment in education into a pronounced divide. A study by Yang et al.^5^ pointed out that, with the gradually widening gap in urban and rural educational resources, the channels for upward mobility for those at the bottom of society are gradually narrowing, leading to the phenomenon where “It is becoming even harder for students to rise from a poor family”.

In the current digital age, the rapid popularity of internet digital technologies is changing every aspect of people’s lives in unprecedented ways. The quick advancement and extensive application of digital technologies have had profound impacts across various fields, and the education industry has also been greatly affected by the wave of digitalization. With the rapid development of digital technology, an increasing number of people have begun to access various educational resources and information through the Internet. Through online platforms, parents can find more abundant educational resources for their kids and can exchange educational experience with education experts and other parents in an online manner. This positive digital access by the Internet has created new opportunities for educational development.

However, with the pervasive diffusion of multifarious educational data via the Internet, the advent of an extensive array of digitized educational information has engendered profound educational anxiety among Chinese parents^6^. Currently, the types and quantity of educational information on the Internet in China are so abundant that they can be described as overflowing. Various types of educational information and advertisements for educational products, such as college planning, college application consulting, and advertisements of online courses, are flooding the Internet. Influenced by these kinds of information on the Internet, parents are more eager for their children to gain an academic advantage in competition with their peers. This can lead parents to seek more educational opportunities for their children and are thus more likely to enroll their children in extracurricular tutoring class. Such behaviors can propel what was originally healthy educational competition towards educational involution. The fever of extracurricular tutoring class brought by the Internet is a social issue in China that needs urgent attention. Yet, the current body of research focusing on this societal concern remains alarmingly scant.

## Literature review

Current research on the impacts of the Internet on education mainly focuses on two aspects: that on the informatization of education, and that on educational expenditure. Regarding the first aspect, studies by Wang et al.^7^ and Hu et al.^8^ found that the Internet has broken the boundaries of traditional classroom education and can effectively achieve information-based development of education. Online teaching can effectively elevate the educational level in regions with fewer educational resources, exerting a positive effect on narrowing the educational divide among regions. Regarding the second aspect, the impact of the Internet on educational expenditure, research by Cui et al.^9^ found that the use of the Internet can significantly increase household educational expenditure. Research by Xu et al.^10^ found that the Internet can increase educational expenditure by increasing family income and raising the importance parents attach to their children's education.

The Internet has broken down the time and space barriers of traditional information dissemination, helping people in accessing all kinds of information. It has built a bridge for parents to connect with the labor demand information of modern society and more advanced educational concepts. With the advent of the digitized intelligent era, low-skilled labor faces the risk of being replaced by intelligent equipment. As parents learn about the trend of social digital development via the Internet, they will inevitably adjust their children’s educational decisions to meet the future needs of the society. In addition, the Internet teems with various types of short videos on college planning, online promotional advertisements from extracurricular tutoring institutions, and paid online courses.

The impact of the Internet on education is a complex and multidimensional process. In addition to directly affecting the informatization of education and educational expenditure, it also involves many issues related to the structure of the education industry, learning methods, educational quality, and educational equity. Previous studies have confirmed that the Internet has a positive impact on changing educational methods and improving learning efficiency. Furthermore, parents have significantly increased their attention to their children's education through the use of the Internet. However, there is currently limited research on the negative effects that the Internet may bring to education.

The proliferation of the Internet and the rise of extracurricular tutoring class often exacerbate issues such as educational intensification and educational anxiety. This is mainly manifested in parents’ continuously increasing educational expenditures for their children, and even a phenomenon of educational comparison among families. As a result, children’s academic pressure continues to increase, focusing solely on academic scores while neglecting their holistic development including their physical and mental health. In the end, this will not be conducive to the healthy development of education.

According to the statistical data of the 2017 China household finance survey (CHFS) data, about 38% of primary and secondary school students in China participated in extracurricular tutoring class in 2017. The average extracurricular tutoring class fee was 1982 yuan per student per year, while the per capita disposable income of Chinese residents in 2017 was 25,974 yuan, indicating that the extracurricular tutoring class fee for a student account for 7.63% of the family’s per capita disposable income. This is undoubtedly a huge educational burden for families with two or three children. However, parents still have motivations to continuously increase extracurricular tutoring class expenditure in case that their children “being left behind at the starting line”^11^. In addition, as China is a society where social interactions are valued, the comparison of extracurricular tutoring class expenditures brought by social interaction can cause educational anxiety^12^, thereby increasing the probability of enrolling their children who were not in extracurricular tutoring class. A study by Fang et al.^13^ confirmed the presence of herd effect in rural education investment, with the education expenditure of surveyed households increasing with the average education expenditure in the village.

The existing research have deeply analyzed the impacts of the Internet on education from different perspectives, but there is still little research combining Internet usage with participation in extracurricular tutoring class. Therefore, by combining Internet usage with participation in extracurricular tutoring class, this paper conducted empirical research on the impacts of Internet usage of parents on enrolling their children in extracurricular tutoring class, meanwhile analyzing the underlying mechanisms of how it works.

## Research hypothesis

Children participate in extracurricular tutoring class can be explicitly modeled as the outcome of several inputs, including credit restrictions^14^^,^ family educational anxiety^15^, and family inputs^16^. Parental inputs are essential factors in the motivation of children in extracurricular tutoring class. In terms of parenting, accurate and up-to-date parenting knowledge is critical to parenting skills improvement and child development^17,18^. Knowledge about optimal parenting is spread primarily through public health platforms and informal networks. Mass media has long been proved to be associated with private household behavior. Keefer and Khemani^19^ find evidence that having access to radio improved parental investment in children’s education. Via the Internet, people can effectively reduce the cost of obtaining information, increase the frequency of social interactions, and optimize the allocation of family resources ^20^. This can profoundly impact family educational expenditures. On the one hand, as an essential source of social networking and information gathering, the internet can potentially influence parental behavior and further affect adolescent in extracurricular tutoring class. The digital access provided by the Internet enables parents to have a more comprehensive understanding of the information related to extracurricular tutoring institutions, extending the channels for parents to understand more information about their educational products. That is, the Internet has expanded the opportunities for parents to browse more information about educational products of the extracurricular tutoring institutions, which can increase the probability of parents enrolling their children in extracurricular tutoring class. On the other hand, with the maturity of Internet technology, precise big data pushes can reach the target population accurately. Extracurricular tutoring institutions can expand their effective target groups through online advertising. This form of Internet-based advertising can also enhance parents' willingness to enroll their children in extracurricular tutoring class. From the analysis of the above two aspects, this paper holds that the use of the Internet can enable parents to obtain more relevant educational products information related to extracurricular tutoring institutions, and it also provides opportunities for extracurricular tutoring institutions to expand their potential customer base through online promotion. Under the combined effects of the above two aspects, the use of the Internet can improve parents’ willingness to enroll their children in extracurricular tutoring class, which means that the use of Internet could increase the probability of parents enrolling their children in extracurricular tutoring class. Therefore, the first research hypothesis H1 was proposed:

H1: Parents’ use of the Internet increases the probability of them enrolling their children in extracurricular tutoring class.

The author posits that whether the use of the Internet will increase the probability of parents enrolling their children in extracurricular tutoring class varies depending on the educational level of the parents. First, parents with a higher level of education more emphasis on their children's education. This is because these parents have obtained relatively high educational returns from their education, so they will want their children to access higher education levels and higher quality educational resources, too, in the hope that their children can also gain higher educational returns. Moreover, parents with a higher level of education have a longer-term perspective in guiding their children’s development. To actively adapt to the future demands of China's digital society for high-quality talents, parents will inevitably increase their investment in their children’s education. Second, parents with a relatively higher level of education have a comparative advantage when using the Internet. They can enhance their proficiency in utilizing the Internet, which enables easier access to relevant educational information. Through the use of Internet, parents can learn about the course’s information, faculty setting, and reputation of different extracurricular tutoring institutions by browsing online platforms and visiting extracurricular tutoring institutions websites. This convenience and transparency brought by the Internet increase the probability of parents with a higher level of education enrolling their children in extracurricular tutoring class. Therefore, this paper argues that the use of the Internet can more likely increase the probability of parents with a higher level of education enrolling their children in extracurricular tutoring class. Based on the above analysis, the paper proposed the second research hypothesis H2:

H2: Through using the Internet, parents with higher level of education are more likely to enroll their children in extracurricular training class.

In the digital age, people have gained broader information channels and social networks through the use of the Internet, which will have a profound impact on people’s decision-making behavior. The Internet makes connections between people more convenient. By using the Internet, parents can have more educational experience exchanges and interactions with other parents, forming an educational social network for information sharing and opinion exchange. This kind of social network is vital in the process of parents deciding whether to enroll their children in extracurricular tutoring class.

Social interaction refers to the process of individuals exchanging information, sharing opinions, and referring to behaviors with others in social network. During the process of social interaction, parents’ decision-making behavior is often influenced by the herd effect, that is, the ideas and behaviors of other members of the same social group. As the Internet has strong social interaction attributes, it makes social interaction more convenient and quicker. People can interact in real-time through social media, video conferences, instant messaging, and other means, and even form multiple interactions in a short period of time, thereby increasing the frequency of social interaction.

When parents engage in social interactions, if they realize that other parents within the same social group have enrolled their children in extracurricular tutoring class, they may feel a form of peer pressure, raising their concerns about “leaving their children behind at the starting line”, thereby enhancing their willingness to enroll their children in extracurricular tutoring class. The impact of herd effect can potentially be amplified through the Internet. Based on the above analysis, the following research hypothesis, H3, was proposed:

H3: The use of the Internet can increase the frequency of parents’ social interaction, which increases the probability of enrolling their children in extracurricular tutoring class.

Through the Internet, parents can learn that more and more jobs and industries are posing higher requirements for higher levels of education in the future digital society. The future job market will have a higher demand for individuals with advanced degrees and professional skills. Those with lower-level qualifications may have difficulty finding solid footing in future digital society. Therefore, by using the Internet, parents will raise their educational expectations for their children so that can their children have a comparative advantage in the future digital society. In addition, whenever the results of China’s college entrance examination are announced, China’s major Internet media widely promotes high-scoring candidates, creating a nationwide climate of admiration for high score achievers. Through media publicity, parents' educational expectations for their children can also be indirectly increased. The higher the educational expectations parents have for their children, thus increasing the probability of enrolling their children in extracurricular tutoring class. Based on the above analysis, the following research hypothesis, H4, was proposed:

H4: The use of the Internet increases parents’ educational expectations for their children, and they are hence more likely to enroll their children in extracurricular tutoring class.

**Figure 1:**
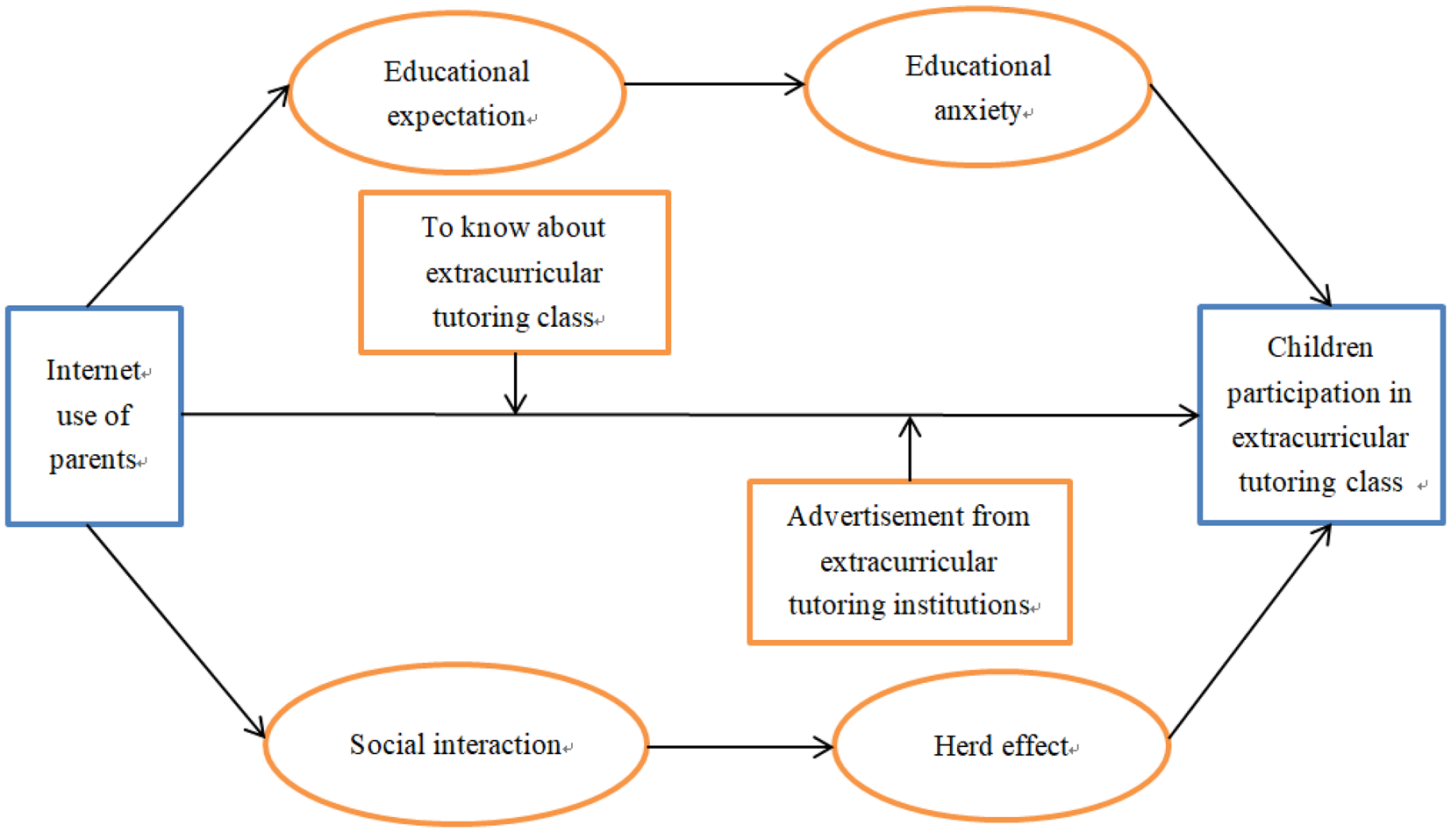
The impact path of Internet use on participation in extracurricular tutoring class.

Figure 1 shows the impact path that the Internet usage of parents enrolling children in extracurricular tutoring class, which is based on the above hypothesis: H1, H3 and H4. According to the above hypothesis, the Internet usage of parents has not only a direct influence on enrolling their children in extracurricular tutoring class, but also has an indirect influence by educational expectation and social interaction.

## Data and model setting

### Data source and descriptive statistics

The data used in this paper is sourced from the China family panel studies (CFPS) for the years 2014, 2016, and 2018 (At present, the CFPS database has been updated to 2020, but the data for 2020 only updated the adult database, and data at the child and household levels have not been released. Therefore, the data for 2020 is not complete as it only includes the level of household heads. That is why this study did not use the latest 2020 samples). This dataset includes family survey data from a total of 25 provinces in China (excluding Hong Kong, Macao, Xinjiang, Tibet, Qinghai, Inner Mongolia, Ningxia, and Hainan), and is highly representative. This study excludes samples of children aged under 6 or over 16, thus retaining samples of children who are currently in the compulsory education stage (attending primary school and junior high school). Since the CFPS database does not provide exact information about the head of the household, this paper follows the general practice of related research and uses the “family finance respondent” as the household head, or parent. To avoid inconsistencies between the household head and the child's parent, samples with household heads under the age of 25 or over the age of 65 are excluded.

The dependent variable of this paper is parents whether to enroll their children in extracurricular tutoring class. As previously mentioned, according to the statistical data from the 2017 China household finance survey (CHFS) data, the extracurricular tutoring class fee for each student in China account for a considerable proportion of the family’s disposable income. Therefore, whether parents enroll their children in extracurricular tutoring class can reflect, to a certain extent, the educational burden brought about by extracurricular tutoring class. In the CFPS dataset, the corresponding survey question is “Does your child attend extracurricular tutoring class?” An affirmative response is denoted with a value of 1, whereas a negative response is indicated by a value of 0. The CFPS data also includes the “Extracurricular tutoring class fee (in RMB)”. This variable is used to replace “Does your child attend extracurricular tutoring class?” as the dependent variable for robustness test.

Parents using the internet improves monetary and goods investment in children, providing resources for children’s development^21^^,^^22^^,^^23^. Therefore, the core explanatory variable is whether parents use the Internet. The survey question in the 2014 CFPS data is “Do you surf the Internet?” and the questions related to the use of Internet in the 2016 and 2018 CFPS data are “Do you use mobile Internet?” and “Do you use computer Internet?”. In the 2014 survey, if the respondent answered “yes”, it is considered that they use the Internet and it is assigned a value of 1, if the answer is “no”, it is assigned a value of 0. For the 2016 and 2018 surveys, the assessment of Internet usage includes two questions: “Do you use mobile Internet?” and “Do you use computer Internet?”. If the respondent answered “yes” to at least one of these questions, it is recorded as 1, indicating that they use the Internet; otherwise, it is recorded as 0.

For control variables, variables related to children’s characteristics, parents’ characteristics, and family characteristics are selected. The analysis also incorporates individual and year dummy variables to control for unobservable factors. The control variables pertaining to children’s characteristics include children’s gender and age. Children’s gender is controlled because families may have educational investment preferences based on the gender of their children. Age is a control variable as the propensity of parents to enroll their children in extracurricular tutoring class may fluctuate depending on the educational stage of the children. For example, as the child’s years of education increase and academic pressure continues to raise, the probability that parents will enroll their children in extracurricular tutoring class also increases, thus necessitating its consideration as a control variable. The control variables for parental characteristics include the parent’s age, gender, level of education, health status, and marital status. Control variables at the family level include family size and income level. Based on the analysis above, this paper holds that the use of the Internet can enhance parents' expected educational level for their children and increase the frequency of social interaction, thereby influencing their children’s participation in extracurricular tutoring class. A mediation effect is used here for mechanism testing. The specific definitions of each variable are shown in Table 1.

**Table 1:** Variable description.

Variable dimension	Variable selection	Variable type	Variable definition
Dependent variable	Extracurricular tutoring class	Whether the children attend extracurricular tutoring class	“Yes” = 1
			“No” = 0
Core explanatory variable	Internet use	Whether the parent uses the internet	“Yes” = 1
			“No” = 0
		Whether the parent accesses mobile internet	“Yes” = 1
			“No” = 0
		Whether the parent accesses computer internet	“Yes” = 1
			“No” = 0
Child characteristic control variables	Child gender	Child’s gender	“Male” = 1
			“Female” = 0
	Child age	Child’s age	Child’s actual age
Parent characteristic control variables	Level of education	Parent’s years of education	Calculated based on the respondent’s level of education
	Health status	Parent’s health status	“Unhealthy” = 1
			“Average” = 2
			“Relatively healthy” = 3
			“Very healthy” = 4
			“Extremely healthy” = 5
	Age	Parent’s age	Logarithm of the age of the head of household
	Marital status	Whether the parent has a spouse	“Married (having a spouse)”
			“Living together” = 1
			“Divorced”
			“Widowed” = 0
	Gender	Parent’s gender	“Male” = 1
			“Female” = 0
	Hukou (household registration) status	Agricultural/non-agricultural hukou	“Agricultural hukou” = 1
			“Non-agricultural Hukou” = 0
	Nature of job	Agricultural/non-agricultural job	“Agricultural Job” = 1
			“Non-agricultural Job” = 0
Family characteristic control variables	Income level	Per capita net income in the family	Logarithm of per capita net income in the family in the past 12 months
	Family size	Number of family members	Logarithm of the number of family members
Variables that influence the mechanism	Educational expectations	Expected level of education for the child	“No need for schooling” = 1
			“Primary school” = 2,
			“Junior high school” = 3
			“High school” = 4
			“Junior college” = 5
			“Undergraduate” = 6
			“Master’s” = 7
			“Doctorate” = 8
	Social interaction	The frequency of social interaction	The proportion of social interaction expenditure to total family income

Table 2 presents grouped data based on whether parents use the internet. It can be observed that in the group of parents who use the internet, 24.96% of them choose to enroll their children in extracurricular tutoring class, while in the group of parents who do not use the internet, only 11.39% of them enroll their children in extracurricular tutoring class. The enrollment rate in the internet usage group is 2.19 times higher than that in another group.

**Table 2:** Descriptive statistics of data for each variable.

Variable Type	N	Mean	Standard deviation	Minimum value	Maximum value
Whether the child attends extracurricular tutoring class	14420	0.165	0.371	0	1
Internet use	14420	0.367	0.482	0	1
Child gender	14419	0.531	0.499	0	1
Child age	14419	10.231	2.878	6	16
Parent’s years of education	12383	6.600	4.674	0	18
Parent’s health status	12383	2.964	1.221	1	5
Parent’s age	12383	44.114	10.394	25	65
Parent’s marital status	12383	0.928	0.258	0	1
Parent’s gender	12383	0.509	0.499	0	1
Hukou status	12383	0.812	0.391	0	1
Nature of job	12383	0.437	0.496	0	1
Per capita net income in the family	12049	59110.610	78318.27	0	2160000
Number of family members	12049	5.147	1.893	2	21

Table 3 shows the descriptive statistics for each variable. A total of 14,420 sample observations were collected from the three periods of data in 2014, 2016, and 2018. According to the descriptive statistical results, 16.5% of the children participated in extracurricular tutoring class, and this proportion has been continuously rising during the research period: 14.6% in 2014, 15.3% in 2016, and 19.4% in 2018. The proportion of samples using the Internet during the research period was 36.7%, with 21.8% of parents using the Internet in 2014, 36.8% in 2016, and 51.6% in 2018, showing a rapid rise.

**Table 3:** Chi-square test of the dependent variable and core explanatory variable.

Variable	Internet usage		
Extracurricular tutoring class		0	1
	0	8116	3948
	1	1043	1313
Pearson chi2(1) = 450.137***			

***Represents the statistical significance levels of 1%.

### Model setting

Given that both the dependent variable and the core explanatory variable are dummy variables, this paper uses a linear probability model for coefficient estimation and Probit model for robustness test. The basic model is set as shown in Eq. (1):

1 $$Class_{it} = \beta_{0} + \beta_{1} Internet_{it} + \mathop \sum \limits_{i = 2}^{11} \beta_{i} Control_{it} + \theta_{i} + \mu_{t} + \varepsilon_{it}$$

The dependent variable, ${Class}_{it}$ in Eq. (1), represents extracurricular tutoring class, i.e., whether parents enroll their children in extracurricular tutoring class. ${\beta }_{0}$ is the constant term. The core explanatory variable is ${Internet}_{it}$, which represents whether parents use the Internet, and ${\beta }_{1}$ is the estimated coefficient of the core explanatory variable. ${Control}_{it}$ is a series of control variables, and ${\beta }_{i}$ is the estimated coefficient of each control variable, reflecting the impact of control variables at the child, parent, and family level on the dependent variable. ${\theta }_{i}$ is the fixed effect of individual parents, ${\mu }_{t}$ is the time fixed effect, and ${\varepsilon }_{it}$ is the heteroscedasticity-robust standard error, following a normal distribution with a mean of 0 and a variance of ${\sigma }_{i}$.

Table 3 presents the Chi-square test of the dependent variable and core explanatory variable. As shown in Table 3, the Pearson Chi-square value with 1 degree of freedom is 450.137, which is significant at the 1% level. This significant result rejects the null hypothesis that there is no correlation between parents’ internet use and children’s enrollment in extracurricular tutoring class. Therefore, it should accept the alternative hypothesis that there is a significant correlation between parents’ internet use and children’s enrollment in extracurricular tutoring class.

## Empirical results and analysis

### Basic regression results

Regarding the model setting in Eq. (1), before performing the basic regression, a model test was done first. This paper used the Hausman test to determine whether Eq. (1) should use a fixed effects model or a random effects model. As shown in Table 4, the Hausman value is 146.31 and is significant at the 1% significance level, indicating that the fixed effects model setting of Eq. (1) is reasonable.

**Table 4:** Hausman test.

	Model (1)	Model (2)
	RE	FE
Internet	−0.008	0.056***
	(0.013)	(0.008)
lnage	0.147***	−0.000
	(0.025)	(0.011)
gender	−0.028	−0.019***
	(0.047)	(0.007)
lnage1	−0.068**	0.072***
	(0.034)	(0.016)
gender1	0.006	−0.016**
	(0.011)	(0.007)
lnedu	−0.006	0.007*
	(0.009)	(0.004)
health	0.002	−0.003
	(0.004)	(0.003)
marriage	−0.051*	0.006
	(0.028)	(0.014)
hk	−0.000	−0.155***
	(0.026)	(0.010)
jobclass	−0.004	0.046***
	(0.015)	(0.008)
lnsize	0.035	−0.042***
	(0.027)	(0.011)
lnfinc	0.009	0.040***
	(0.006)	(0.004)
cons	−0.016	−0.293**
	(0.157)	(0.084)
N	12049	12049
Hausman-value	146.31***	

***, ** and *represent statistical significance levels of 1, 5 and 10% respectively. The figures in parentheses are normal standard errors.

Table 5 presents the basic regression results of this study. In Table 5, $lnage$ and $gender$ are control variables for children’s characteristics, representing the natural logarithm of the child's age and the gender of children, respectively. $lnage1$*, *$gender1$*, *$lnedu$*, *$health$*, *$marriage$*, *$hk$*, and *$jobclass$ are control variables for parent’s characteristics, representing the natural logarithm of the parent’s age, parent’s gender, natural logarithm of parent’s years of education, parent’s health status, marital status, hukou, and nature of job, respectively. $lnsize$ and $lnfinc$ are control variables for the family characteristics, which are the of the number of family members and the natural logarithm of per capita net family income, respectively; cons are the constant term corresponding to ${\beta }_{0}$ in Eq. (1).

**Table 5:** Regression results of Internet usage on participation in extracurricular tutoring class.

	Model (1)	Model (2)	Model (3)	Model (4)	Model (5)
Internet	0.136***	0.107***	0.107***	0.066***	0.054***
	(0.007)	(0.007)	(0.007)	(0.009)	(0.009)
lnage			0.020**	0.000	−0.004
			(0.010)	(0.010)	(0.010)
gender			−0.017***	−0.016***	−0.019***
			(0.006)	(0.006)	(0.006)
lnage1				0.061***	0.072***
				(0.015)	(0.015)
gender1				−0.018***	−0.017**
				(0.007)	(0.007)
lnedu				0.010***	0.005*
				(0.003)	(0.003)
health				−0.002	−0.003
				(0.003)	(0.003)
marriage				0.004	0.007
				(0.012)	(0.013)
hk				−0.174***	−0.162***
				(0.012)	(0.012)
jobclass				0.064***	0.049***
				(0.007)	(0.008)
lnsize					−0.047***
					(0.010)
lnfinc					0.041***
					(0.004)
cons	0.114***	0.124***	0.087***	0.018	−0.362***
	(0.004)	(0.004)	(0.022)	(0.068)	(0.079)
Individual fixed effect	NO	YES	YES	YES	YES
Time fixed effect	NO	YES	YES	YES	YES
Adjusted R^2^	0.031	0.089	0.090	0.145	0.151
F-value	394.94***	228.44***	79.24***	60.35***	59.15***
N	14420	14419	14419	12383	12049

***, ** and *represent statistical significance levels of 1, 5 and 10% respectively. The figures in parentheses are robust standard errors.

If the estimated coefficient ${\beta }_{1}$ of the core explanatory variable in Eq. (1) is significantly greater than 0, it indicates that parents significantly increase the likelihood of enrolling their children in extracurricular tutoring class by using the internet. Conversely, if ${\beta }_{1}$ is significantly less than 0, it indicates that parents significantly decrease the likelihood of enrolling their children in extracurricular tutoring class by using the internet. If ${\beta }_{1}$ is not significantly different from 0, it indicates that there is no relationship between parents using the internet and enrolling their children in extracurricular tutoring class. Moreover, due to the advantages of the LPM model, the magnitude of the estimated coefficient ${\beta }_{1}$ directly represents the marginal impact of the core explanatory variable on the dependent variable.

Models from (1) to (5) in Table 5 are structured on the foundation of single variable regression, with a gradual introduction of control variables to test the robustness of the estimated coefficients between the core explanatory variable and the dependent variable. Models (1) and (2) are single variable regressions, only including the core explanatory variable and the dependent variable. Model (1) does not include individual and time fixed effects and pertains to correlation regression analysis, while Model (2) includes individual and time fixed effects. Models (3), (4), and (5) sequentially add control variables for children’s characteristics, parents’ characteristics, and family characteristics based on Model (2). The estimated results of Eq. (1) are shown in Model (5) of Table 4. As is shown in Model (5) of Table 4, it indicates that the probability of parents enrolling their children in extracurricular tutoring class could increase by 5.4% through the use of the Internet. In addition, in Table 4, from Models (1) to (5), the impact of Internet use on participation in extracurricular tutoring class is significantly positive at the 1% significance level. After sequentially adding individual and time fixed effects, children’s characteristics, parent’s characteristics, and family characteristics variables, the estimated coefficient of the core explanatory variable remains significantly positive, indicating that the model is robust. Thus, H1 is validated.

In the control variables, the estimated coefficient for child gender is significantly negative, indicating that females are more likely to participate in extracurricular tutoring class compared to males. The impact of household head age on whether children participate in extracurricular tutoring class is significantly positive; as the household head’s age increases, the likelihood of enrolling children in extracurricular tutoring class also increases. The estimated coefficient for household head gender is significantly negative, suggesting that when the household head is female, there is a higher likelihood of children enrolling in extracurricular tutoring class compared to when the household head is male. The estimated coefficient for household head education years is significantly positive, indicating that higher levels of education for the household head are associated with a higher likelihood of enrolling children in extracurricular tutoring class. Household registration status significantly affects participation in extracurricular tutoring class, with parents from urban areas having a higher likelihood of enrolling their children. The number of family members and per capita net income are significantly negative and positive, respectively, showing that lighter family burdens increase the likelihood of children participating in extracurricular tutoring class.

### Heterogeneity regression analysis

#### Heterogeneity analysis of parents’ education level

A grouped regression analysis was conducted according to different years of education received by the parents. As shown in Table 2, the average years of education for parents is 6.6 years. Taking the mean value of education years as a benchmark and rounding down, parents’ education level is divided into primary school and below, and above primary school. The grouped regression results are shown in Table 6. Model (1) represents the group of parents with 6 years or less of education, and Model (2) represents the group of parents with more than 6 years of education. In Model (1), the coefficient estimate of the core explanatory variable is positive, but not significantly different from 0. The positive impact of Internet use on participation in extracurricular tutoring class is not quite significant in this group. The core explanatory variable in Model (2) is significantly positive at the 1% significance level. Parents with relatively higher education levels are more significantly influenced by the Internet and are more likely to enroll their children in extracurricular tutoring class. Hypothesis H2 is validated.

**Table 6:** Heterogeneity regression results of parents’ education level.

	Model (1)	Model (2)
	Primary school and below	Above primary school
Internet	0.026	0.042***
	(0.019)	(0.013)
lnage	0.011	−0.003
	(0.015)	(0.017)
gender	0.008	−0.038***
	(0.009)	(0.010)
lnage1	0.110***	0.042***
	(0.023)	(0.027)
gender1	−0.016	−0.020*
	(0.010)	(0.011)
health	−0.001	−0.002
	(0.004)	(0.005)
marriage	0.014	0.016
	(0.016)	(0.026)
hk	−0.066	−0.167***
	(0.040)	(0.014)
jobclass	0.040***	0.050***
	(0.015)	(0.012)
lnsize	−0.024	−0.082***
	(0.016)	(0.016)
lnfinc	0.012**	0.067***
	(0.006)	(0.008)
cons	−0.418***	−0.436***
	(0.126)	(0.138)
Individual fixed effect	YES	YES
Time fixed effect	YES	YES
Adjusted R^2^	0.054	0.159
F-value	13.63***	49.24***
N	4125	7924

***, ** and *represent statistical significance levels of 1, 5 and 10% respectively. The figures in parentheses are robust standard errors.

### Regression on the influencing mechanism

In Table 7, Model (1) is the regression of Internet use on social interaction. The regression result of Model (1) shows that the impact of Internet use on social interaction is significantly positive at the 1% significance level, indicating that Internet use could significantly increase the frequency of social interaction. Model (2) is the regression of social interaction on participation in extracurricular tutoring class. The estimated coefficient is significantly positive at the 1% significance level. This means that the higher frequency of social interaction can significantly increase the probability of parents enrolling their children in extracurricular tutoring class. Model (3) incorporates both the mediator variable (social interaction) and the core explanatory variable (internet usage) into the regression equation. The results indicate that the effects of the mediator variable and the core explanatory variable on the dependent variable are both significantly positive at the 1% significance level, and it means that there is partial mediation effect.

**Table 7:** Regression results of the influence mechanism.

	Model (1)	Model (2)	Model (3)	Model (4)	Model (5)	Model (6)
	Social interaction	Class	Class	Expectation	Class	Class
Internet	0.992***		0.051***	0.075***		0.042***
	(0.337)		(0.009)	(0.027)		(0.008)
Interaction		0.001***	0.001***			
		(0.000)	(0.000)			
Expectation					0.017***	0.016***
					(0.003)	(0.003)
Control variables	YES	YES	YES	YES	YES	YES
Individual fixed effect	YES	YES	YES	YES	YES	YES
Time fixed effect	YES	YES	YES	YES	YES	YES
Adjusted R^2^	0.033	0.151	0.153	0.049	0.152	0.154
F-value	32.82***	82.68***	78.83***	31.45***	60.51***	57.73***
N	11497	11354	11343	12090	11995	11982

***represents statistical significance levels of 1%. The figures in parentheses are robust standard errors.

The advent of the Internet has bridged geographical gaps, fostering enhanced communication among individuals. Internet adoption lowers communication, transportation, and search costs which boost human capital formation, trade between countries, and economic productivity^24–26^. With the Internet, people have interacted with each other more frequently. Research by Zhou & Fan. ^20^ has also confirmed that the use of the Internet increases the frequency of social interaction within families ^22^. The increase in social interaction significantly promotes the formation of a “herd effect” in educational investment among different families. Xu et al.^12^ found that families with frequent social interactions are influenced by neighboring families, thereby increasing their educational investment. Considering these findings, this paper argues that the Internet has increased the frequency of social interaction between parents. The increase in social interaction has formed a “herd effect” of participation in extracurricular tutoring class. Parents that were not originally enrolled their children in extracurricular tutoring class are influenced by neighboring parents who have enrolled their children in extracurricular tutoring class, which in turn increase the probability of parents enrolling their children in extracurricular tutoring class. Thus, H3 is validated.

In Table 7, Model (4) represents the regression of the impact of Internet use of parents on children’s educational expectations. The regression results of Model (4) show that the impact of Internet use on educational expectations is significantly positive at the 1% statistical significance level. This means that Internet use significantly enhances parents’ educational expectations for their children. Model (5) represents the regression of educational expectations on participation in extracurricular tutoring class. The regression result of Model (5) shows that the impact of educational expectations on participation in extracurricular tutoring class is significantly positive at the 1% statistical significance level, suggesting that the elevation of parents’ educational expectations for their children significantly increases the probability of enrolling their children in extracurricular tutoring class. Model (6) is the regression that includes internet usage and educational expectation, and based on the regression result it reveals that there exists partial mediation effect among internet usage, education expectation and participation in extracurricular tutoring class.

The Internet has broken the barriers of time and space in information dissemination, increasing people’s access to information. This helps parents understand advanced educational concepts and talent demands in modern society. At the same time, the intensifying situation of education competition in society is also constantly transmitted to parents through the Internet, causing education anxiety. Under the combined effect of these two factors, educational expectations of parents for their children are significantly raised through the use of the Internet. The increase in parents' educational expectations for their children will inevitably intensify educational investment. A study by Li et al.^27^ found that when parents realize their children need to have higher human capital levels, they will increase their educational investment in their children. Based on empirical results and theoretical analysis, the author argues that parents significantly enhance their educational expectations for their children through the use of the Internet, thereby increasing the probability of parents enrolling their children in extracurricular tutoring class. Hence, research hypothesis H4 is confirmed.

### Robustness test

#### Endogeneity

In the basic regression shown in Table 4, there may be a bias in the estimated results due to endogeneity. Although various control variables are added in the basic regression, there are still unobservable variables in the residual term that may cause estimation bias in the basic regression results; meanwhile, there is a certain reverse causal relationship between Internet use and participation in extracurricular tutoring class. Parents who intend to enroll their children in extracurricular tutoring class may learn to use the Internet to collect relevant information. Therefore, to solve the endogeneity problem, this paper used the instrumental variable (IV) method and propensity score matching (PSM) estimation for endogeneity. The IV regression as shown in Table 8, and PSM as shown in Table 9.

**Table 8:** Results of the instrumental variable regression.

	First-stage regression Internet		Second-stage regression class
Internet			0.474***
			(0.208)
IV	0.006***		
	(0.001)		
Control variables	YES		YES
Individual fixed effect	YES		YES
Time fixed effect	YES		YES
Cragg-Donald Wald F statistic	39.465[16.38]		
Kleibergen-Paap rk LM statistic	37.751***		
N	12,049		

***, ** and *represent the statistical significance levels of 1, 5 and 10% respectively. Robust standard errors are shown in parentheses, and the critical value of Stock Yogo weak identification test at 10% level is shown insquared brackets.

**Table 9:** PSM estimation.

	Matching method	Treatment group	Control group	ATT	Standard error	T-value
Class	Neighbor matching	0.247	0.158	0.089***	0.014	6.17
	Radius matching	0.247	0.150	0.097***	0.012	8.15
	Kernel matching	0.247	0.151	0.096***	0.012	8.03

***represents the statistical significance levels of 1%.

After referring to the research of Ning et al.^28^^,^ Huang et al.^29^^,^ and Zhao et al.^30^, this paper used the 2014, 2016 and 2018 Internet penetration rate at the regional level as the instrumental variable, and this variable is from the China Internet network information center (CNNIC). We choose this instrumental variable because Internet penetration rate data at the regional level represents the development of regional Internet infrastructure, with the more extensive Internet infrastructure construction, the more likely to access the Internet for the individuals. In addition, the Internet penetration rate at the regional level cannot influence the decisions of parents whether enrolling their children in extracurricular tutoring class. This meets the requirement that the instrumental variable can only affect the dependent variable through the core explanatory variable. The regression results are shown in Table 8.

Table 8 shows the estimation results after using the instrumental variable regression. As can be seen from the regression result of first-stage regression, the relationship between the instrumental variable and the core explanatory variable is significantly positive at the 1% significance level. After using the instrumental variable regression, the estimated coefficient of the core explanatory variable still maintains the same sign as the benchmark regression results in Table 5. The relationship between the core explanatory variable and the dependent variable is positive at the 1% significance level. After testing the instrumental variable, the null hypothesis of weak instruments and under-identification are rejected. Therefore, the results of the basic regression in Table 4 are valid and H1 is validated again.

The estimated results of PSM are shown in Table 9, where parents who use the internet are classified as the treatment group, and those who do not use the internet are classified as the control group. Table 8 employs three matching methods: neighbor matching, radius matching, and kernel matching. Neighbor matching is conducted with a 1:4 ratio for with-replacement sampling matching, and radius matching uses a radius of 0.05, and kernel matching sets the bandwidth at 0.06. Across these three matching methods, the coefficient differences between the treatment and control groups are significantly positive at the 1% level of significance. Parents who use the internet are significantly more likely to enroll their children in extracurricular education and training than those who do not use the internet, confirming once again the positive impact of internet use on participation in extracurricular education and training.

#### Changing the estimation model

As the dependent variable is a dummy variable, a probit model was used for re-estimation. However, as the probit model is a random-effects model, it does not accommodate individual fixed effect at the same level as the microdata. Therefore, we substituted individual fixed effect with provincial fixed effect. Table 10 shows the estimation result after changing the model. After changing the estimation method, the relationship between Internet use and participation in extracurricular tutoring class remains significantly positive at the 1% significance level. Internet use has a significant positive impact on participation in extracurricular tutoring class, which is consistent with the basic regression results in Table 5. Furthermore, the estimated coefficients of each control variable in Table 8 are consistent with the signs of the estimated coefficients of each control variable in Table 5, indicating that the model setup and estimation results are robust.

**Table 10:** Probit model estimation results.

	Probit
Internet	0.230***
	(0.041)
lnage	−0.031
	(0.054)
gender	−0.085***
	(0.031)
Lnage1	0.110***
	(0.023)
Gender1	−0.099***
	(0.032)
lnedu	0.037*
	(0.020)
health	−0.005
	(0.013)
marriage	0.043
	(0.070)
hk	−0.500***
	(0.041)
jobclass	0.261***
	(0.039)
lnsize	−0.221***
	(0.050)
lnfinc	0.238***
	(0.020)
cons	−4.550***
	(0.435)
Provincial fixed effect	YES
Time fixed effect	YES
Wald chi2	1779.44***
N	12042

*** and **represent statistical significance levels of 1 and 5%, respectively. The figures in parentheses are normal standard errors.

#### Replacing the dependent variable

A robustness test was performed by replacing the dependent variable in Table 4’s regression, from (“Does your child attend extracurricular tutoring class?”) to “Extracurricular tutoring class fee (in RMB)”. The regression results are shown in Table 11. Models (2) to (5) sequentially add individual and time fixed effects, control variables for children’s characteristics, parent’s characteristics, and family characteristics, based on the single-variable correlation regression in Model (1). After replacing the dependent variable, the estimated coefficient between the core explanatory variable and the dependent variable remains significantly positive, indicating that the results of regression in Table 4 are robust.

**Table 11:** Robustness test for the replaced dependent variable.

	Model (1)	Model (2)	Model (3)	Model (4)	Model (5)
Internet	2.263***	1.146***	1.162***	0.730***	0.655***
	(0.070)	(0.061)	(0.061)	(0.077)	(0.078)
lnage			0.675***	0.607***	0.543***
			(0.091)	(0.093)	(0.095)
gender			−0.072	−0.061	−0.085
			(0.053)	(0.054)	(0.055)
lnage1				0.486***	0.612***
				(0.140)	(0.143)
gender1				−0.272***	−0.237***
				(0.058)	(0.058)
lnedu				0.158***	0.112***
				(0.032)	(0.032)
health				−0.025	−0.032
				(0.023)	(0.023)
marriage				0.171	0.252**
				(0.119)	(0.124)
hk				−1.181***	−1.096***
				(0.084)	(0.085)
jobclass				0.658***	0.531***
				(0.069)	(0.071)
lnsize					−0.583***
					(0.093)
lnfinc					0.266***
					(0.032)
cons	1.505***	1.868***	0.365*	-0.820	−2.984***
	(0.040)	(0.033)	(0.212)	(0.633)	(0.717)
Individual fixed effect	NO	YES	YES	YES	YES
Time fixed effect	NO	YES	YES	YES	YES
Adjusted R^2^	0.093	0.401	0.406	0.446	0.451
F-value	1045.61***	353.59***	137.35***	94.55***	84.53***
N	10155	10152	10152	8762	8540

***, ** and *represent the statistical significance levels of 1, 5 and 10% respectively. The figures in parentheses are robust standard errors.

## Conclusions and policy recommendations

This paper used the 2014, 2016, and 2018 CFPS statistical data to empirically analyze the impacts of Internet use on participation in extracurricular tutoring. The empirical results show that parents significantly increase the probability of enrolling their children in extracurricular tutoring class through the use of the Internet. According to the basic regression result in Table 4, it shows that the probability of parents enrolling their children in extracurricular tutoring class could increase by 5.4% through the use of the Internet. After a series of robustness tests, including instrumental variable regression, probit regression, and replacing the dependent variable and core explanatory variable, the impact of Internet use on participation in extracurricular tutoring class remains significantly positive. Heterogeneity analysis reveals that parents with higher levels of education are more inclined to enroll their children in extracurricular tutoring class. The mechanism analysis shows that parents can increase the frequency social interaction and raise their educational expectations for their children through the use of the Internet, thereby increasing the probability of enrolling their children in extracurricular tutoring class.

Based on the conclusions of this research, the following policy recommendations were proposed: (1) Governments at all levels in China should establish a more comprehensive after-school education service system during the compulsory education stage. After-school education services can play a vital role in reducing the cost of extracurricular tutoring class. However, due to the limited experience in after-school education services in primary and secondary schools at all levels at this stage, the after-school education service system needs further improvement, and the quality-of-service delivery must be enhanced. As such, the government should endeavor to refine the implementation rules of after-school education services. This will not only reduce the educational expenditure but also utilize spare time to cultivate a more holistic educational experience for students. (2) The government can increase education investment, promote education reform, establish evaluation mechanisms, strengthen teacher training, and enhance home-school cooperation to increase the supply of in-school education resources and provide higher-quality education services. Specifically, this includes increasing education funding to improve school infrastructure and teaching equipment, promoting innovation in educational content and teaching methods, establishing comprehensive evaluation mechanisms to monitor education quality, enhancing teacher training and career development support, fostering close cooperation between schools and families to jointly focus on students’ educational growth, and providing better educational environments and opportunities for students. (3) Because household educational investment on their children can affect the intergenerational human capital accumulation of individual family, and furthermore affect the economic sustainable development of the whole society^31^. Therefore, educational involution is markedly acute in urban areas of China, while rural areas are grappling with a dearth of educational resources and a shortage of teaching faculty. The long-term shortage of educational resources in rural areas can potentially perpetuate the intergenerational transmission of poverty. Therefore, on the policy level, more educational resources should be tilted toward rural areas. The surplus educational resources in urban areas should be introduced into rural areas through policy measures, enabling rural children to access a conductive educational environment and more equitable educational opportunities.

## Limitations and future improvement

This paper is based on CFPS data to explore the impact of Internet use on the participation in extracurricular tutoring class. However, there are still some limitations in this study. One of the main limitations is lack of precise data. In this study, we use the Internet use as the core explanatory variable to explore the influence on participation in extracurricular tutoring class, but this is an imprecise variable to measure the effect of Internet use. We are expected to use the time allocation that parents use Internet to browse some educational information, which can precisely reflect on the Internet use on education. However, there is lack of some relevant data, which is the main limitation in this study. Another limitation is also that due to lack of data, we cannot obtain the amount of extracurricular tutoring class that parents enroll their children in. In reality, there are lots of China’s parents enroll their children in more than one kind of extracurricular tutoring class, such as mathematical, physics and chemistry. If we use the amount of extracurricular tutoring class that children participation in as the explained variable, the results of regression would be more convinced. Therefore, the future improvement of this study is to use other micro dataset to deeply explore the impact of Internet use on the participation in extracurricular tutoring class.

Based on the CFPS data used in this study, we noticed that the proportion of participation in extracurricular tutoring class gradually increased from 2014 to 2018. The average cost of extracurricular education and training reached 1112.56 RMB, which is a significant education expense for individual families. During a press conference in April 2020, the then Chinese Premier Li Keqiang mentioned, “Out of 1.4 billion Chinese, 600 million have a monthly income of less than 1000 RMB.” Such high expenditures on extracurricular education may not promote fairness in the allocation of Chinese education resources, increase the burden of family education investment, and lead to economic education anxiety. Furthermore, China’s educational system has long been focused on selective exams for advancement. The pressure of academic advancement persists from high school to undergraduate and graduate levels. Alongside academic pressure, parents are inevitably inclined to invest more family resources in education. This exam-oriented system undoubtedly adds more psychological pressure to families. Therefore, in future research, the authors will explore education anxiety from multiple dimensions, including economic pressure and the pressure of selective exam-oriented educational systems, to provide a comprehensive understanding of this phenomenon.

## Data Availability

The datasets generated and/or analysed during the current study are available in the [China Family Panel Studies, CFPS] repository, [https://www.isss.pku.edu.cn/cfps/?CSRFT=KNWG-4TLA-9IA7-RUI0-69IO-JR5N-34BB-RSI3].

## Abbreviations

CFPS: China family panel studies
CHFS: China household finance survey
